# Hematogenous Dissemination of Breast Cancer Cells From Lymph Nodes Is Mediated by Tumor MicroEnvironment of Metastasis Doorways

**DOI:** 10.3389/fonc.2020.571100

**Published:** 2020-10-26

**Authors:** Anouchka Coste, George S. Karagiannis, Yarong Wang, Emily A. Xue, Yu Lin, Mihaela Skobe, Joan G. Jones, Maja H. Oktay, John S. Condeelis, David Entenberg

**Affiliations:** ^1^Department of Anatomy and Structural Biology, Einstein College of Medicine/Montefiore Medical Center, New York, NY, United States; ^2^Department of Surgery, Einstein College of Medicine/Montefiore Medical Center, New York, NY, United States; ^3^Gruss-Lipper Biophotonics Center, Einstein College of Medicine/Montefiore Medical Center, New York, NY, United States; ^4^Integrated Imaging Program, Einstein College of Medicine/Montefiore Medical Center, New York, NY, United States; ^5^Department of Oncological Sciences and Tisch Cancer Center, Icahn School of Medicine at Mount Sinai, New York, NY, United States; ^6^Department of Pathology, Einstein College of Medicine/Montefiore Medical Center, New York, NY, United States; ^7^Department of Epidemiology and Population Health, Einstein College of Medicine/Montefiore Medical Center, New York, NY, United States

**Keywords:** breast cancer, lymph node, blood vessel, lymphatic vessel, cancer cell dissemination, tumor microenvironment of metastasis (TMEM)

## Abstract

In primary breast tumors, cancer cells hematogenously disseminate through doorways in the vasculature composed of three-cell complexes (known as Tumor MicroEnvironment of Metastasis) comprising a perivascular macrophage, a tumor cell overexpressing the actin-regulatory protein Mammalian Enabled (Mena), and an endothelial cell, all in direct physical contact. It has been previously shown that once tumor cells establish lymph node metastases in patients, TMEM doorways form in the metastatic tumor cell nests. However, it has not been established if such lymph node-TMEM doorways actively transit tumor cells into the peripheral circulation and on to tertiary sites. To address this question in this short report, we used a mouse model of lymph node metastasis to demonstrate that TMEM doorways: (1) exist in tumor-positive lymph nodes of mice, (2) are restricted to the blood vascular endothelium, (3) serve as a mechanism for further dissemination to peripheral sites such as to the lungs, and (4) their activity can be abrogated by a pharmaceutical intervention. Our data suggest that cancer cell dissemination via TMEM doorways is a common mechanism of breast cancer cell dissemination to distant sites and thus the pharmacological targeting of TMEM may be necessary, even after resection of the primary tumor, to suppress cancer cell dissemination.

## Introduction

Cancer cell intravasation, a critical step in the metastatic cascade, does not occur along the entirety of cancer-associated vasculature, but is restricted instead to specialized intravasation doorways, called Tumor MicroEvironment of Metastasis (TMEM). TMEM doorways are composed of a perivascular macrophage, a tumor cell highly expressing the actin-regulatory protein Mammalian Enabled (Mena), and an endothelial cell, all in direct physical contact with each other (1–3). Prior studies have shown that the number of TMEM doorways in primary breast tumors is prognostic of distant metastasis, independent of lymph node status, and other currently used prognosticators (4–6). Mechanistically, perivascular macrophages in TMEM doorways are capable of secreting vascular endothelial growth factor-A (VEGFA) in a high concentration in a Tie2-dependent manner. As a consequence, VEGFA-mediated breakdown of the underlying endothelial-specific junctions results in transient, localized vascular permeability, which allows for the passing of highly-invasive cancer cells into the circulation (7, 8). Indeed, the targeted pharmacological inhibition of TMEM activity by using the selective Tie2 inhibitor rebastinib, has been shown to eliminate cancer cell dissemination and metastasis *in vivo* (9, 10).

TMEM-mediated vascular permeability and breast cancer cell intravasation have been observed through multiphoton intravital imaging (IVI), and are determined to last approximately 20 min not only in primary tumors (8), but also in newly formed TMEM doorways of established metastatic lesions in lungs (11). Moreover, we have recently reported that TMEM assembly occurs in established lymph node metastasis of breast cancer patients, and that TMEM doorways are always associated with blood, and not with lymphatic, vessels in both primary tumors and their respective lymph node metastases (12). Based on our previous findings of TMEM doorway development in metastatic lymph nodes of breast cancer patients (12), here, we conclusively demonstrate that “secondary-site” TMEM doorways are functional, are actively disseminating tumor cells into the circulation, and that this process can be stopped with a pharmacological intervention.

## Materials and Methods

### Cell Culture

The cell line MDA-MB-231-SORE6-dsCopGFP was generated from parental MDA-MB-231 cells, as previously described (13) and was used because it gives a high frequency of spontaneous lymph node metastasis, obviating the need for direct injection of cells into the afferent lymphatics. This model allows for the investigation of the behavior of cancer cells and secondary sites that have been educated by the presence of the primary tumor (14–16). The Dendra2 MDA-MB-231-SORE6-dsCopGFP cell line was generated by inducing Dendra2-MDA-MB-231 cells (17) with SORE6-dsCopGFP viruses, as has been previously described in Tang et al. (13). The cells were cultured in DMEM (Invitrogen, Carlsbad, CA, USA) with 10% fetal bovine serum (FBS) and 50U penicillin/50 μg streptomycin per mL, except for the Dendra2 cell media, supplemented with 250 μg/ml geneticin (Invitrogen).

### Mouse Models

All procedures were conducted in accordance with the National Institutes of Health regulations and approved by the Albert Einstein College of Medicine animal care and use committee. The fluorescent cell line MDA-MB-231-SORE6-dsCopGFP has been described previously (13), and can develop spontaneous lymph node metastasis within 2–3 months after orthotopic transplantation in immunodeficient SCID (NCI, Frederick, MD, USA) or MacBlue/Rag2^−/−^ mice. MacBlue/rag2^−/−^ mice were generated by crossing Rag2^−/−^ mice (Rag2^−/−^ model RAGN12, Taconic) with Tg(Csf1r^*^-GAL4/VP16,UAS-ECFP)1Hume/J mice (Stock No: 026051, The Jackson Laboratory). To generate the orthotopic MDA-MB-231-SORE6-dsCopGFP xenografts, a total of 0.5 × 10^6^ cells per animal were re-suspended in sterile PBS with either 20% collagen I (BD Biosciences, Franklin Lakes, NJ, USA) for SCID mice or 50% matrigel for MacBlue/Rag2^−/−^ mice, and injected in the lower left mammary fat pad. The mice were allowed to grow primary tumors for 3 months, and those with positive lymph nodes were randomly allocated to experimental groups. To suppress TMEM doorway activity in primary and/or secondary tumor sites, we performed treatments with the small molecule Tie2 inhibitor rebastinib, as previously described (9, 10). In particular, rebastinib (provided by Deciphera Pharmaceuticals), was reconstituted at a concentration of 10 mg/ml in 0.4% hydroxypropyl methylcellulose (HPMC). Each mouse in the experimental group received *p.o*. doses of 10 mg/kg rebastinib (100 μl total volume), twice per week, for 3 weeks. The control (vehicle-treated) group received *p.o*. 100 μl of HPMC. For the photoconversion experiments, Dendra2-expressing MDA-MB-231-SORE6-dsCopGFP cells were used to generate xenograft tumors in immunodeficient SCID mice.

### Photoconversion

Mice bearing primary breast tumors were prepared as described above using Dendra2-expressing 231-SORE6-dsCopGFP tumor cells (Dendra2^+^ tumor cells). Positive inguinal lymph nodes that were spatially separated from the primary tumors by at least 3 mm were identified by the transdermal observation of green fluorescence using an epi-fluorescence stereoscope (Olympus, SZX16) equipped with a mercury lamp (Lumen Dynamics, X-Cite Series 120Q). An aluminum foil mask that shielded the primary tumor from exposure to light, and allowed illumination only of the inguinal lymph node, was prepared and affixed to the anesthetized animal. Photoconversion was accomplished by 8 min of continuous transdermal illumination using a 405 nm filter (Chroma, D405/30M), with the lamp set to its highest intensity setting, and with the stereoscope additionally set to its highest magnification (11.5x, which produced an ~2 mm illumination spot). Complete photoconversion of the lymph nodes was verified in two mice by immediate excision of the converted node and comparison of the red fluorescence signal before and after a final round of photoconversion. Absence of photoconversion within primary tumors was verified in the same two mice also by immediate excision of the primary tumors and measurement of its red fluorescence signal. Twenty-four hours after photoconversion, mice were sacrificed, and lungs were excised and prepared for frozen sectioning. Sections were stained for DAPI and imaged on a Pannoramic P250 digital whole slide scanner.

Identification of photoconverted cells was accomplished by sequential application of three custom developed Visiopharm apps to the digital whole slide scans. The first app identified the boundaries of the tissue. This was accomplished by applying a median blur with an 11 × 11 pixel kernel to the red, green, and blue channels (to smooth out the individual nuclei and cells) and then subtracting both the red and the green channels from the blue channel to eliminate from consideration any regions with a high level of background. Next, all photoconverted and unconverted single cells were identified by processing the green channel with a Poly Gradient (with an Order of 1 and Filter Size of 3), applying a non-linear stretch to the gray levels (in order to make separation from background easier), and then thresholding the signal. This algorithm identified all photoconverted and unconverted cells since photoconversion always leaves an appreciable amount of unconverted protein. Size based filters ensured that small fragments of cells (<50 μm^2^) and groupings of cells that would be larger than recently disseminated single cells or tumor cell clusters (>2,500 μm^2^, or groups of ~16 cells) were eliminated. The outlines of these cells were then used as ROIs in the final app that identified which of the cells identified with the second app were photoconverted (red) cells. This was accomplished by employing the same algorithm as the second app, but this time processing the red channel.

### TMEM Immunohistochemistry

Immunohistochemistry of TMEM Supplementary Figure 1 for all samples was performed on an autostainer, as described (5, 6, 18) with only mild modifications. Specifically, the mouse lymphatic vessel TMEM (LV-TMEM) protocol substituted D2-40 for endomucin (1:50 dilution; abcam) to specifically detect lymphatic endothelial cells. The macrophage (IBA-1) and Pan-Mena markers remained the same with our previously described blood vessel TMEM (BV-TMEM) protocol. LV-TMEM and BV-TMEM scores were evaluated and compared by one pathologist in a total of nine metastatic lymph node samples. The scores for both BV-TMEM and LV-TMEM were reported as number of TMEM doorways per 10 high-power fields (HPFs), as described (1, 5, 6).

### Extravascular Dextran Analysis

Assessment of TMEM-mediated vascular permeability was performed, using multichannel-immunofluorescence (IF) in an FFPE section, aligned with a sequential BV-TMEM triple immunohistochemistry section, as described (19). TMEM activity is expressed as extravascular dextran (%), which is calculated as the area covered by extravascular dextran divided by total area in each image. In this assay, TMEM activity is signaled as the robust and inhomogeneous (due to its directional release and rapid clearance) expression of dextran around a blood vessel (i.e., endomucin-expressing profile) (8, 9, 19).

### CD206^+^ Macrophage Immunofluorescence

Immunofluorescence staining and analysis of the macrophage markers CD206 and IBA1 were performed in established lymph node metastases as described for primary breast tissues (8).

### Statistical Analysis

All two-group comparisons were assessed using the non-parametric Mann–Whitney *U*-test. The graphs were plotted using means and standard deviation (SD). The GraphPad Prism 7.01 was used for graphing and statistical hypothesis testing.

## Results and Discussion

### TMEM Intravasation Doorways Are Newly Formed From Blood Vasculature of Established Lymph Node Metastasis in Mice

**Figure 1:**
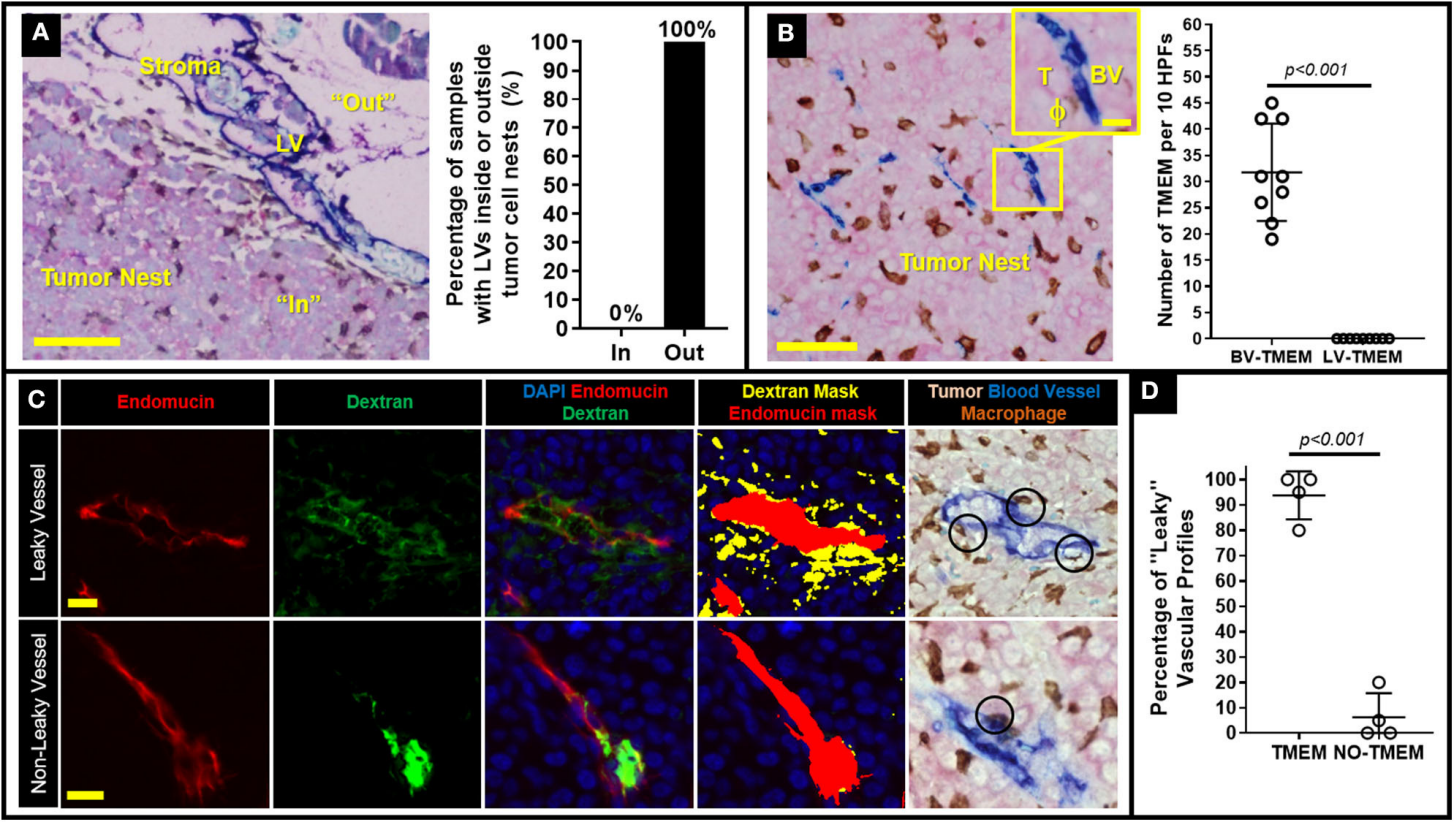
Decreasing TMEM doorway function suppresses TMEM-mediated vascular permeability and cancer cell intravasation from established lymph node metastasis. **(A)** Left: Lymph node metastasis taken from a mouse with an MDA-MB-231 primary breast tumor and stained for lymphatic vessel TMEM (LV-TMEM). Pink = Tumor cells (stained for panMena), Brown = macrophages (stained for CD68), Blue = lymphatic vessels (stained for D2-40). Lymphatic vessels (LV) are only seen in the tumor stroma (“Out”) and not in the tumor nest (“In”) and no lymphatic vessel TMEM (LV-TMEM) were identified in the tumor nests. Bar = 100 μm. Right: Frequency (%) of lymphatic vessels inside or outside the tumor nests in established lymph node metastases in mice. **(B)** Left: Lymph node metastasis taken from a mouse with an MDA-MD-231 primary breast tumor and stained for blood vessel TMEM (BV-TMEM). Pink = Tumor cells (stained for panMena), Brown = macrophages (stained for CD68), Blue = blood vessels (stained for CD31). Bar = 50 μm. Inset shows a magnified image of a BV-TMEM and its constituent cells T = tumor cell, BV = blood vessel, ϕ=macrophage. Inset Bar = 10 μm. Right: Quantification of the number of BV-TMEM and LV-TMEM found in the tumor nests in 10 high power fields of view (HPF). Right: Distribution of scores for BV-TMEM and LV-TMEM in the tumor nests of mouse lymph nodes with established metastases (Mann–Whitney *U*-test, *p* < 0.001). **(C)** Multichannel immunofluorescence-based measurement of local blood vessel leakiness to a high molecular weight (155 kD) dextran using fluorescent antibody staining against endomucin (red; first column) and dextran (green, second column). The merged image (third column), along with DAPI (blue) enables quantification of the amount of extravascular dextran assessment shown as thresholded masks in the fourth column (red = blood vessel, yellow = extravascular dextran). Fifth column shows a sequential slide stained for BV-TMEM and aligned to show the same vessels. Black circles indicate TMEM doorways identified by pathologists. The two corresponding slides were cut in an interval of ~10 μm, hence the slight difference in the alignment of the profiles. Top row shows representative images of leaky vessels and bottom row shows non-leaky vessels. **(D)** Percentage (%) of “leaky” (i.e., with abundant extravascular dextran) blood vessels associated with TMEM, or not associated with TMEM in lymph node metastases (*N* = 4).

We have previously demonstrated in breast cancer patients that once tumor cells escape the primary tumor site and develop metastatic nodules in regional lymph nodes, TMEM doorways form exclusively on blood (not lymphatic) vessel endothelium (12). We thus reasoned that such secondary TMEM doorways may function as intravasation gateways for cancer cell dissemination to tertiary metastatic sites. To study this possibility, we generated a xenograft model which spontaneously develops palpable lymph node metastasis as early as ~3 months after orthotopic injection of 0.5 × 10^6^ 231-SORE6 tumor cells in the mammary fat pads of Rag2^−/−^ recipients (13). In concordance with human breast cancer patients (12), we observed lymphatic vessels (LVs) within the stroma at the periphery of established lymph node metastases in 100% of the cases examined, but not within the tumor nests themselves (Figure 1A). Therefore, lymphatic vessel TMEM (LV-TMEM) doorways could not be detected in the tumor nests of established lymph node metastases. However, blood vessel TMEM (BV-TMEM) was found in the tumor nests of all cases of breast tumors examined (Figure 1B). It should be noted that prior studies using fixed tissue (20) and intravital imaging (8) suggest that cancer cells disseminate via vasculature present in the tumor nests and not in the peritumoral stroma. As such, we focused on BV-TMEM doorways found within the tumor mass of established lymph node metastasis. Collectively, our observations demonstrate that TMEM doorways in mice, as in humans (12), are exclusively associated with blood vessels and show that tumor cells in established lymph node metastases are unlikely to utilize intratumoral LV-TMEM doorways for re-dissemination to tertiary sites.

### BV-TMEM Doorways in Established Lymph Node Metastasis Are Associated With Increased Vascular Permeability

To investigate whether BV-TMEM of established lymph node metastases are active sites of cancer cell re-dissemination to tertiary sites, we first assessed a critical hallmark of TMEM function, TMEM-mediated vascular permeability, using a previously-developed TMEM activity assay that measures extravasation of 155-kDa dextran, conjugated to tetramethylrhodamine (TMR), into the tumor tissue (9, 19). In this assay, the molecular weight of the dextran is chosen as 155-kDa because it has been shown using intravital imaging that 155-kDa dextran leaks from tumor vasculature exclusively due to TMEM-dependent permeability, and not from other forms of vascular leakiness (8, 19). Immunohistochemical staining of BV-TMEM was then co-aligned with sequential IF-stained slides to evaluate the presence or absence of extravascular dextran associated with TMEM doorways, as previously described in detail (19). High resolution imaging of individual vessels shows vasculature with abundant extravascular dextran (Figure 1C, top row: “leaky blood vessels”) and vasculature with minimal or no extravascular dextran (Figure 1C bottom row: “non-leaky blood vessel”). Interestingly, after selecting 20–30 leaky vascular profiles for each mouse [based on tissue size, degree of vascularization, quality of endomucin staining, and feasibility of tissue alignment (19)], co-localization with the TMEM stained slide (Figure 1C, rightmost image) showed that >95% of the leaky vessel profiles were associated with at least one TMEM doorway in lymph node metastases (Figure 1D). This critical observation demonstrates that TMEM doorways in established lymph node metastases are associated with increased vascular permeability, further suggesting that secondary site TMEM doorway could serve as active doorways for cancer cell re-dissemination to tertiary sites.

### Tie2 Inhibition Suppresses TMEM-Mediated Vascular Permeability in Established Lymph Node Metastases

**Figure 2:**
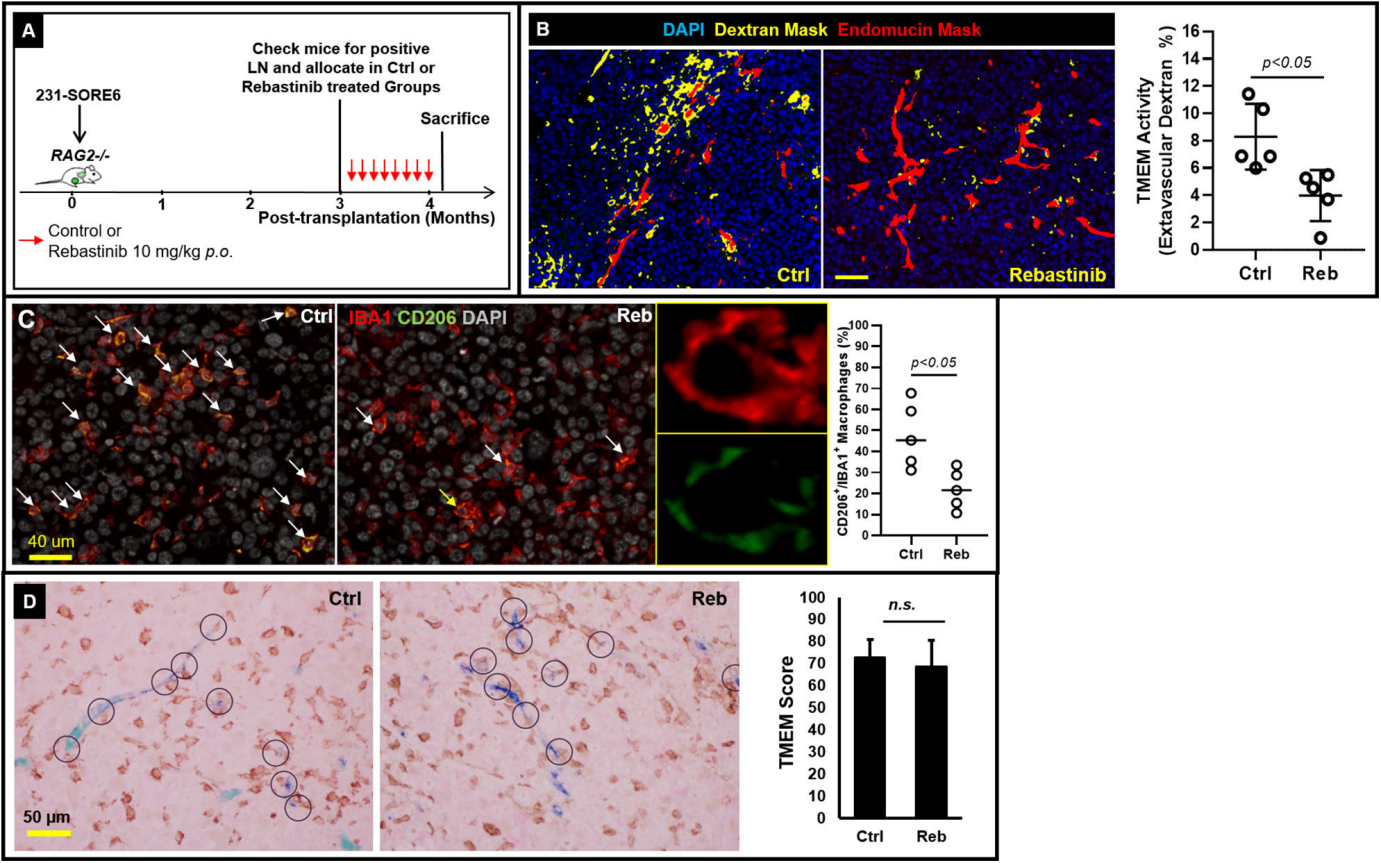
TMEM doorways in established lymph node metastases mediate vascular permeability for cancer cell re-dissemination to tertiary sites. **(A)** Experimental design of control and rebastinib-treated animals with established lymph node metastases. **(B)** Left: Representative examples of the extravascular dextran assessment using multichannel immunofluorescence imaging in 231-SORE6 mice treated with either vehicle (left) or rebastinib (right). Right: Quantification of extravascular dextran area (%) in control and rebastinib-treated mice (Mann–Whitney *U*-test, *p* < 0.05) shows a significant reduction in TMEM-mediated vessel leakiness upon treatment with rebastinib (Reb). **(C)** Left: Formalin-fixed paraffin-embedded sections of lymph node tissue with established metastatic nodules was stained for the macrophage specific marker IBA1 and the M2-polarization marker CD206, and the CD206+IBA1+ macrophages (pointed with the arrows) were scored as a proportion of the total IBA1+ macrophages in vehicle-treated (Ctrl; left panel) and rebastinib treated (Reb; right panel) 231-SORE6 xenografts. Nuclei were counterstained with DAPI. Snapshots are representative images from the experimental groups. Middle: Magnification of the macrophage shown with the yellow arrow in the right image on the rebastinib-treated example image on the left confirms co-expression of IBA1 and CD206 on the same macrophage. Right: Quantification of CD206+IBA1+ macrophages in the images shown on the left. **(D)** Left: Representative examples of formalin-fixed paraffin-embedded sections of lymph node tissue with established metastatic nodules stained for TMEM triple-IHC stain and scored in vehicle-treated (Ctrl; left panel) and rebastinib-treated (Reb; right panel) 231-SORE6 xenografts. Right: Quantification of TMEM scores in the images shown in the Left. n.s., non-significant.

Having shown that lymph node metastases are capable of assembling blood vessel TMEM doorways Figures 1A–D *de novo*, we subsequently sought to unravel whether such secondary TMEM doorways are active in disseminating tumor cells hematogenously. Previously, the pharmacological Tie2 inhibitor, rebastinib, was established as a potent and highly selective inhibitor of the Tie2 kinase, with minimal or absent off-target effects (10). Since Tie2 kinase activity is required for TMEM activity, rebastinib is a selective and potent suppressor of TMEM activity, thereby eliminating cancer cell intravasation and dissemination (9, 10). To substantiate that vascular leakiness is TMEM-dependent in metastatic lymph nodes as well, we compared the degree of TMEM-associated vascular leakiness between vehicle- and rebastinib-treated mice. Indeed, mice that received treatment with rebastinib in a dosing scheme (Figure 2A) previously documented to inhibit TMEM function in primary tumors (9, 10) demonstrated a significant reduction (Mann–Whitney *U*-test; *p* < 0.05) of extravascular dextran (Figure 2B). Moreover, rebastinib treatment significantly (*p* < 0.05) reduces recruitment of proangiogenic CD206^+^ tumor-associated macrophages in the microenvironment of established lymph node metastases (Figure 2C), which support a prometastatic phenotype (21), which additionally involves streaming of tumor cells toward TMEM and TMEM doorway-dependent vascular permeability and cancer cell dissemination (10). However, rebastinib treatment does not significantly alter the assembly of TMEM doorways in lymph nodes, as assessed by TMEM scoring between vehicle- and rebastinib-treated mice (Figure 2D). Overall, these data indicate that decreased vascular leakiness upon rebastinib treatment in established lymph node metastases is the direct result of TMEM function suppression, rather than decrease in the physical assembly of TMEM doorways.

### TMEM Doorways in Established Lymph Node Metastases Mediate Cancer Cell Re-dissemination to Tertiary Sites

**Figure 3:**
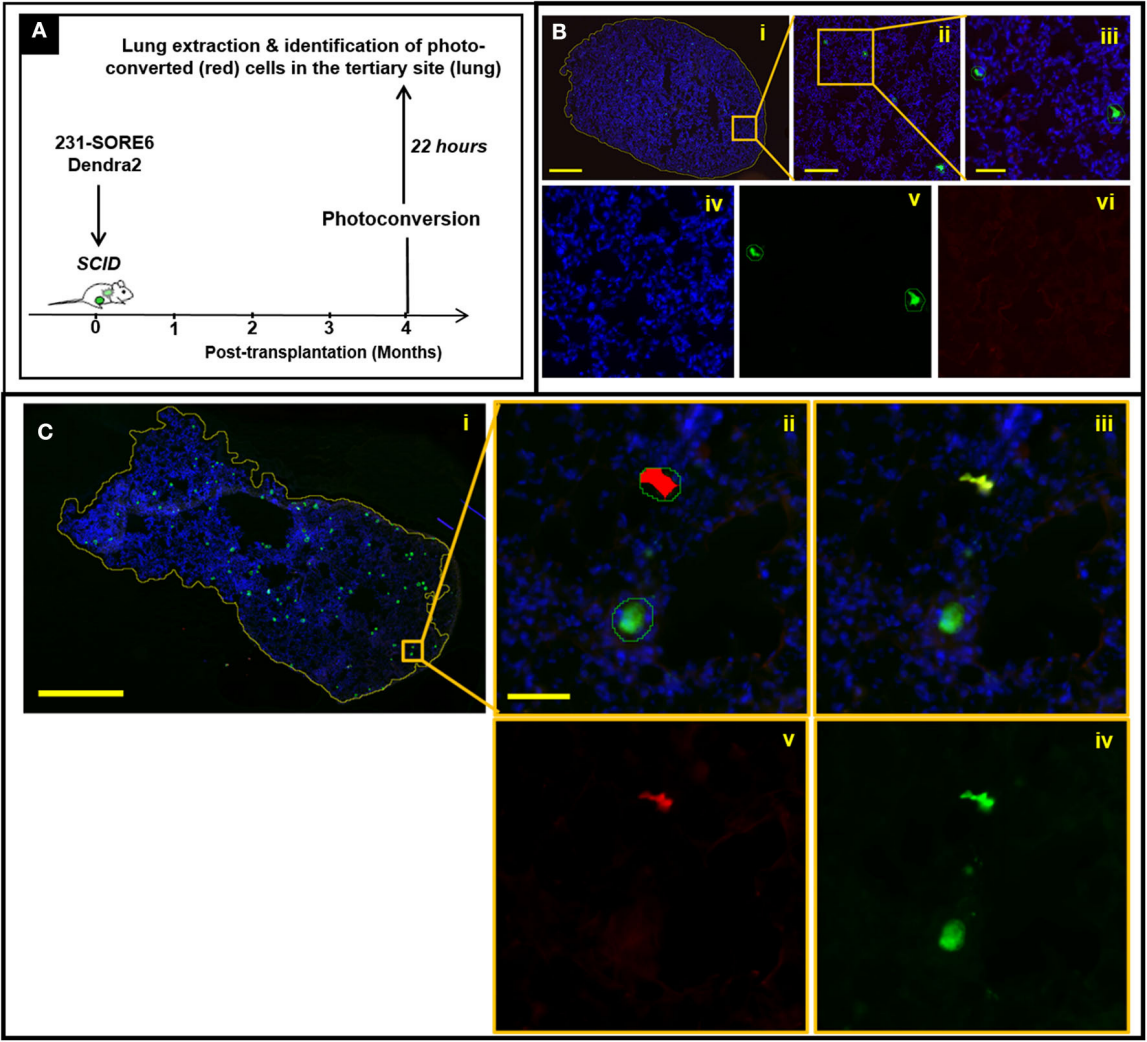
Disseminated tumor cells (DTCs) in the lungs originate from established lymph node metastases in breast cancer xenografts. **(A)** Experimental design of disseminated tumor cell tracking analysis (mice sacrificed 22 h after photoconversion). **(B)** Absence of photoconverted cells in fixed frozen sections of lung tissue from mice with negative lymph nodes. **(i)** Fixed-frozen sections of lung tissue were scanned on a digital whole slide scanner and then loaded into Visiopharm. The first of three apps identifies the boundaries of the tissue (yellow outline around tissue section). Scale bar = 1 mm. (ii) Zoomed image of the region indicated by the orange box in (i). The second app identifies both unconverted and photoconverted cells (outlined with green lines). Scale bar = 200 μm. (iii) Further zoom-in of the region indicated by the orange box in (ii). Scale bar = 50 μm. (iv–vi) Individual DAPI (iv), green (v), and red (vi) fluorescence channels of the image shown in (iii). The third and final app identifies which of these cells are photoconverted, as indicated by the red overlay in (vi). The identification of cells as tumor cells is confirmed by the green fluorescence of the unconverted cells (v), while yellow fluorescence (red plus green) would be expected in the photo-converted cells because they show both Dendra2 colors. The isolated green channel in (v) shows both photoconverted and unconverted tumor cells. The isolated red channel in (vi) shows only cells which have been photoconverted. As expected however, no photoconverted cells are observed in mice with lymph nodes that are negative for tumor cells, indicating that photoconversion of tumor cells in the lymph node is indeed specific to the lymph nodes. **(C)** Presence of photoconverted cells in fixed frozen sections of lung tissue from mice with positive lymph nodes. (i) Fixed-frozen sections of lung tissue were scanned on a digital whole slide scanner and then loaded into Visiopharm. The first of three apps identifies the boundaries of the tissue (yellow outline around tissue section); (ii) Zoomed image of the region indicated by the orange box in (i); the second app identifies all photoconverted and unconverted cells (outlined with green lines) and the third and final app identifies which of these cells are photoconverted (indicated by the red overlay); (iii) Identification of cells as tumor cells is confirmed by the green fluorescence of the unconverted cells, and the yellow fluorescence (red plus green) of the converted cells; (iv) The isolated green channel shows all tumor cells; (v) The isolated red channel shows only cells which have been photoconverted.

We have previously shown that TMEM-mediated vascular permeability in primary breast tumors is associated with cancer cell intravasation (8). As such, observations shown in Figure 2 are consistent with the expectation that TMEM doorways in lymph node metastases are also capable of mediating cancer cell intravasation. To confirm this, we employed a method that allowed us to determine the site of origin and time of dissemination of cancer cells that have metastasized to distant sites, such as to the lung. This strategy (Figure 3A) utilizes the photoconvertible fluorescent protein, Dendra2, as described previously (22–24). Dendra2 is a green-emitting fluorescent protein that can be converted into additionally emitting red light by exposure to ultraviolet light (25). In particular, we first stably expressed the fluorescent protein Dendra2, then orthotopically implanted 231-SORE6-Dendra2 tumor cells into syngeneic mice, and after 4 months of tumor growth, we used a stereoscope equipped with an epifluorescent lamp and a 405 nm excitation filter to convert Dendra2^+^ cancer cells from green-to-red fluorescence while limiting photoconversion to only the metastatic lymph nodes. After 24 h, we determined whether photoconverted disseminated tumor cells (DTCs) that had been photoconverted in the metastatic lymph nodes, disseminated successfully and appeared in a tertiary site, in particular the lungs (Figures 3B,C). To confirm specificity of this assay, we first photoconverted lymph nodes from animals without established metastatic nodules in their lymph nodes and performed assessment of photoconverted Dendra2^+^ cells in extracted lung tissues. Although Dendra2^+^ tumor cells were identified in lung sections, suggesting primary tumor cell origin, no photoconverted cells were noticed among them Figure 3B, suggesting that photoconversion is indeed specific to the lymph nodes. We then proceeded to photoconversion experiments in lymph nodes from animals with established metastatic nodules in their lymph nodes and evaluated the presence of red fluorescent DTCs (Figure 3C, i-v) in the lung clearly proves that these cells originated from lymph node metastases, and not from other sites (including the primary tumor), and that they arrived at the lung after the time-point of photoconversion. Overall, these observations indicate that breast cancer cells are capable of hematogenous dissemination from established lymph node metastases via a TMEM-mediated mechanism to other distant organs.

## Conclusions

In the current study, we demonstrate, for the first time, that metastatic nodules in regional lymph nodes in mice can assemble TMEM doorways (similar to those observed in the primary breast tumor microenvironment) that are capable of hematogenous cancer cell dissemination to distant sites. Our data, especially those illustrated in Figures 1C,D and Figures 2B,D, clearly reveal that TMEM doorways exist in tumor-bearing sites other than the primary tumor sites, are potentially active, and can be pharmacologically inhibited. These observations further suggest that the overall TMEM biology is a component of the metastatic cascade, significant, and critical enough to be recapitulated in multiple steps of the cascade, including primary tumors (8, 9), regional metastatic foci in lymph nodes (current study), and even distant metastatic foci (11).

Quite surprisingly, lymphatic vessels were absent from the tumor parenchyma of established lymph node metastases, indicating that cancer cell re-dissemination primarily follows a hematogenous route for re-dissemination. These observations are in agreement with previously published work showing that distant seeding from lymph node metastases occurs via a hematogenous route (26–28), although a direct link to a specialized cancer cell intravasation doorway was not shown at the time. In further support of our current findings, a recent study has examined the evolutionary history of metastatic breast cancer, and revealed minimal, but not absent, seeding from axillary lymph nodes (29). Taken together, these observations suggest that distant breast cancer metastases may rise from disseminated tumor cells (DTCs) of diverse origins, including dissemination from primary tumor and re-dissemination from lymph nodes.

Tumor-associated macrophages (TAMs) with involvement in cancer cell dissemination and metastasis have been previously described in both the primary tumor microenvironment whereby they participate in TMEM assembly and function, and secondary tumor microenvironments (i.e., lungs), whereby they develop metastasis-supporting niches (2, 3, 8, 30–40). The lymph node microenvironment in this context is rather unique, as it includes at minimum five distinct macrophage subtypes with specific lineage markers and unique immune functions (41, 42). The contribution of these macrophage subtypes in the processes of cancer cell dissemination and metastasis via lymph nodes has not been addressed in the current study, but it should represent a critical and urgent future direction, because deciphering the underlying mechanisms of lymph node metastasis will assist toward the development of treatment modalities to eliminate further metastatic dissemination to tertiary sites. As an insight toward this direction, we demonstrated in the current study, that Tie2 inhibition via rebastinib, significantly alters TMEM activity through the recruitment of fewer CD206^+^TIE2^+^ TAMs supporting the activity of TMEM doorways. However, more studies are needed, especially in the context of depleting diverse macrophage subsets to clarify their individual and collective contributions to cancer cell dissemination from lymph nodes to tertiary metastatic sites.

In this brief research report, we have shown that pharmacological inhibition of TMEM-function can effectively suppress TMEM-dependent vascular permeability and hematogenous dissemination from established lymph node metastasis. As such, rebastinib can suppress TMEM activity, irrespective of whether TMEM doorways are located in the primary tumor site, or a tumor-positive lymph node. Overall, the conclusions collectively drawn from these observations suggest that Tie2 inhibitors may have additional clinical utility against systemic dissemination following the surgical removal of the primary tumor.

## Data Availability Statement

The raw data supporting the conclusions of this article will be made available by the authors, without undue reservation.

## Ethics Statement

The animal study and all procedures were conducted in accordance with the National Institutes of Health regulations, and approved by the Albert Einstein college of Medicine animal care and use committee.

## Author Contributions

Conceptualized study: GK, MO, JJ, JC, and DE. Designed and/or performed experiments: AC, GK, YW, EX, YL, MS, JJ, MO, JC, and DE. Wrote the article: GK and DE. All authors read, modified and approved the final article.

## Conflict of Interest

The authors declare that the research was conducted in the absence of any commercial or financial relationships that could be construed as a potential conflict of interest.
