# Supplementary figures and images for: Hematogenous Dissemination of Breast Cancer Cells From Lymph Nodes Is Mediated by Tumor MicroEnvironment of Metastasis Doorways
Source: Front Oncol. 2020 Oct 26;10:571100. doi: 10.3389/fonc.2020.571100 (PMC7649363; doi:10.3389/fonc.2020.571100)

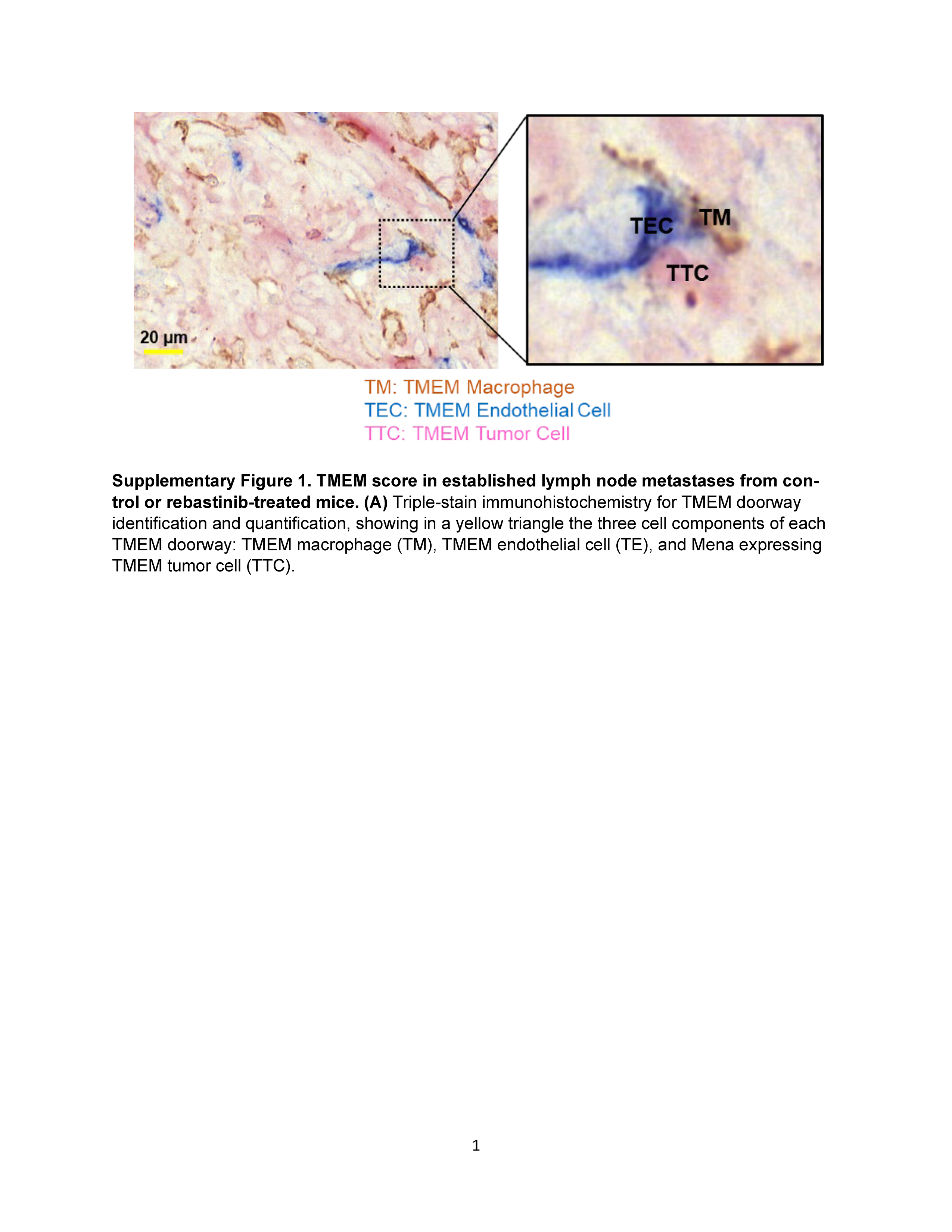

Supplement: Supplementary file 1 [file Image_1.tif]
